# Genome-wide association study and polygenic risk score analysis of esketamine treatment response

**DOI:** 10.1038/s41598-020-69291-6

**Published:** 2020-07-28

**Authors:** Qingqin S. Li, Ewa Wajs, Rachel Ochs-Ross, Jaskaran Singh, Wayne C. Drevets

**Affiliations:** 1 Neuroscience Therapeutic Area, Janssen Research and Development, LLC, 1125 Trenton-Harbourton Road, Titusville, NJ USA; 2 Neuroscience Therapeutic Area, Janssen Research and Development, LLC, Beerse, Belgium; 3 Neuroscience Therapeutic Area, Janssen Research and Development, LLC, La Jolla, CA USA; 4 Present Address: Neurocrine Biosciences, Inc., 12780 El Camino Real, San Diego, CA 92130 USA

**Keywords:** Genome-wide association studies, Depression

## Abstract

To elucidate the genetic underpinnings of the antidepressant efficacy of S-ketamine (esketamine) nasal spray in major depressive disorder (MDD), we performed a genome-wide association study (GWAS) in cohorts of European ancestry (n = 527). This analysis was followed by a polygenic risk score approach to test for associations between genetic loading for psychiatric conditions, symptom profiles and esketamine efficacy. We identified a genome-wide significant locus in *IRAK3* (*p* = 3.57 × 10^–8^, rs11465988, β = − 51.6, SE = 9.2) and a genome-wide significant gene-level association in *NME7* (*p* = 1.73 × 10^–6^) for esketamine efficacy (i.e. percentage change in symptom severity score compared to baseline). Additionally, the strongest association with esketamine efficacy identified in the polygenic score analysis was from the genetic loading for depressive symptoms (*p* = 0.001, standardized coefficient β = − 3.1, SE = 0.9), which did not reach study-wide significance. Pathways relevant to neuronal and synaptic function, immune signaling, and glucocorticoid receptor/stress response showed enrichment among the suggestive GWAS signals.

## Introduction

Esketamine nasal spray has been shown to have rapidly-acting antidepressant effects in patients with treatment resistant depression (TRD) and in patients with major depressive disorder (MDD) at imminent risk for suicide^1–8^. Predictors for conventional oral antidepressant treatment outcome including sociodemographic, symptom profiles, genetics, and clinical comorbidities were systematically reviewed by Perlman et al.^9^ In a small clinical study assessing the antidepressant efficacy of ketamine, a racemate consisting of two enantiomers, *R*- and *S*-ketamine, it was recently reported that body mass index (BMI) was associated with the remission rate, with greater BMI being associated with greater remission rate^10^. BMI and clinical comorbidities are influenced by both genetic and environmental factors. Genetic loading of such traits provides an objective way of measuring the relationship between these and other predictors with antidepressant treatment response.

In studies assessing individual genetic factors, the brain-derived neurotrophic factor (*BDNF*) Val66Met allele was reported to impair basal and ketamine-stimulated synaptogenesis in prefrontal cortex in vitro^11^, and a significant genetic association between Val66Met and ketamine treatment outcome at 4 h post treatment was reported in a candidate gene study of small sample size^12^. A more recent study further suggested that the *BNDF* Val66Met polymorphism may influence the improvement in suicide ideation following ketamine infusion in a sample of depressed participants from Taiwan^13^. In general, however, genetic associations with MDD disease susceptibility outcome reported in relatively small candidate gene studies have proven difficult to replicate in studies of larger samples^14^. Therefore, studies of genetic effects influencing antidepressant treatment outcome may particularly benefit from the use of genome-wide association analysis (GWAS) approaches in clinical trials of larger patient samples. Here, we assessed the genetic contributions to esketamine treatment response from patients with TRD who participated in two Phase III trials testing the efficacy and safety of esketamine, using both a genome-wide association analysis and a polygenic risk score (PRS) approach.

## Results

Esketamine treatment response outcome was assessed at the 4 week study endpoint using one continuous variable (percent change from baseline in the Montgomery–Asberg Depression Rating Scale (MADRS) score ) and two dichotomized variables (responder status, defined by a reduction of ≥ 50% on the MADRS, and remission status, defined by achieving a final MADRS score of < 12). The demographic and clinical characteristics of study participants are summarized in Table 1 and Supplemental Table 1. Participants of the randomized TRANSFORM-3 study were recruited from an elderly population and had a lower remission rate than participants of the open-labelled SUSTAIN-2 study. Gender and concomitant medication proportions were comparable between remitters and non-remitters. After controlling for study, the baseline demographic characteristics (age and baseline BMI) were comparable between remitters and non-remitters. As expected from the clinical literature, remitters had lower baseline depression symptom severity score than non-remitters.

**Table 1:** Characteristics of study participants comparing remitters from non-remitters.

	Remitters (n = 255)	Non-remitters (n = 272)	p-value
**Mean (SD)**			
Age*	50.6 (13.8)	53.4 (13.5)	0.424
Baseline BMI*	28.1 (5.6)	28.3 (5.8)	0.742
Baseline MADRS score*	29.7 (4.7)	33.0 (4.7)	6.36E-13
**N (%)**			
Gender, female	153 (60.0)	175 (64.3)	0.349
Study			7.34E−05
TRANSFORM-3	10 (3.9)	39 (14.3)	
SUSTAIN-2	245 (96.1)	233 (85.7)	
**Concomitant antidepressant medications**			0.782
DULOXETINE	90 (35.3)	87 (32.0)	
ESCITALOPRAM	78 (30.6)	80 (29.4)	
SERTRALINE	42 (16.5)	52 (19.1)	
VENLAFAXINE XR	45 (17.6)	52 (19.1)	
None		1 (0.4)	

*p-value reported is based on type III test statistics controlling for study.

**Figure 1:**
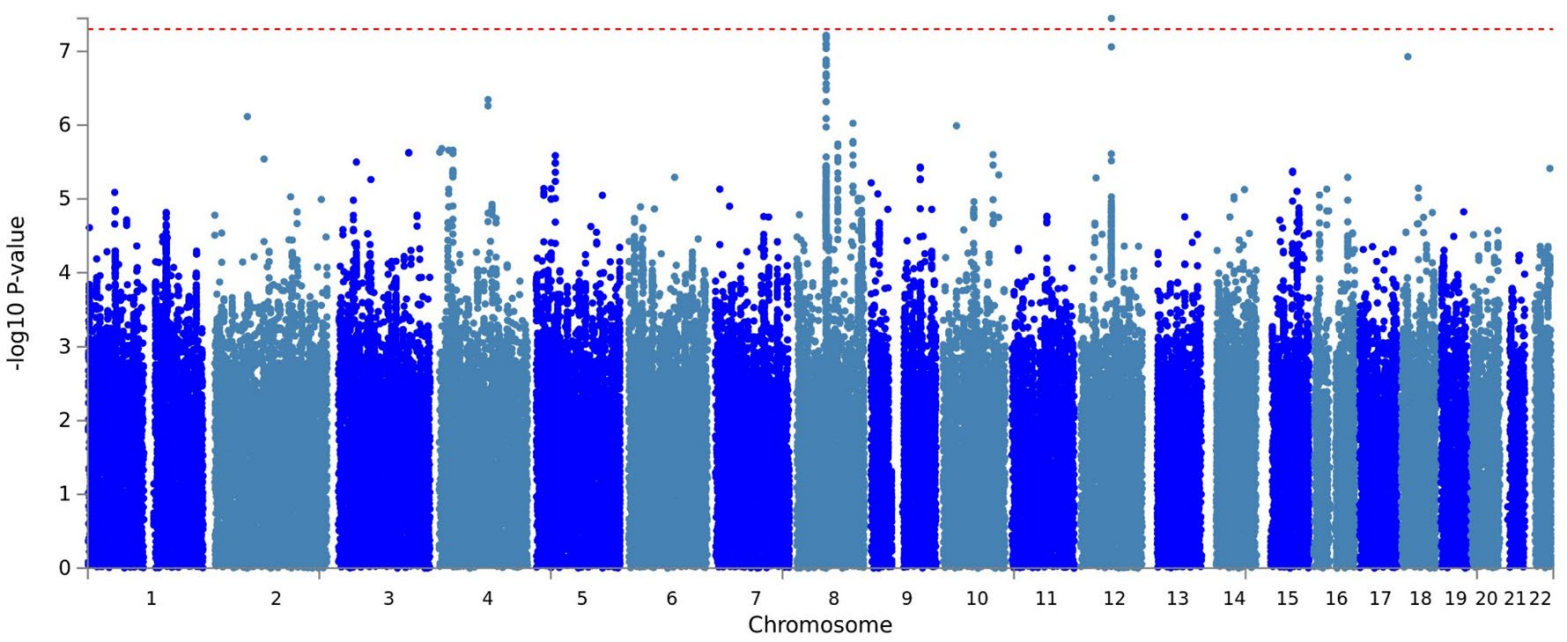
Manhattan plot of the esketamine efficacy endpoint (percentage change of MADRS score at endpoint compared to baseline) generated via FUMA^51^ v1.3.5e (https://fuma.ctglab.nl/). The red dotted line indicates genome-wide significance threshold of 5 × 10^–8^.

**Figure 2:**
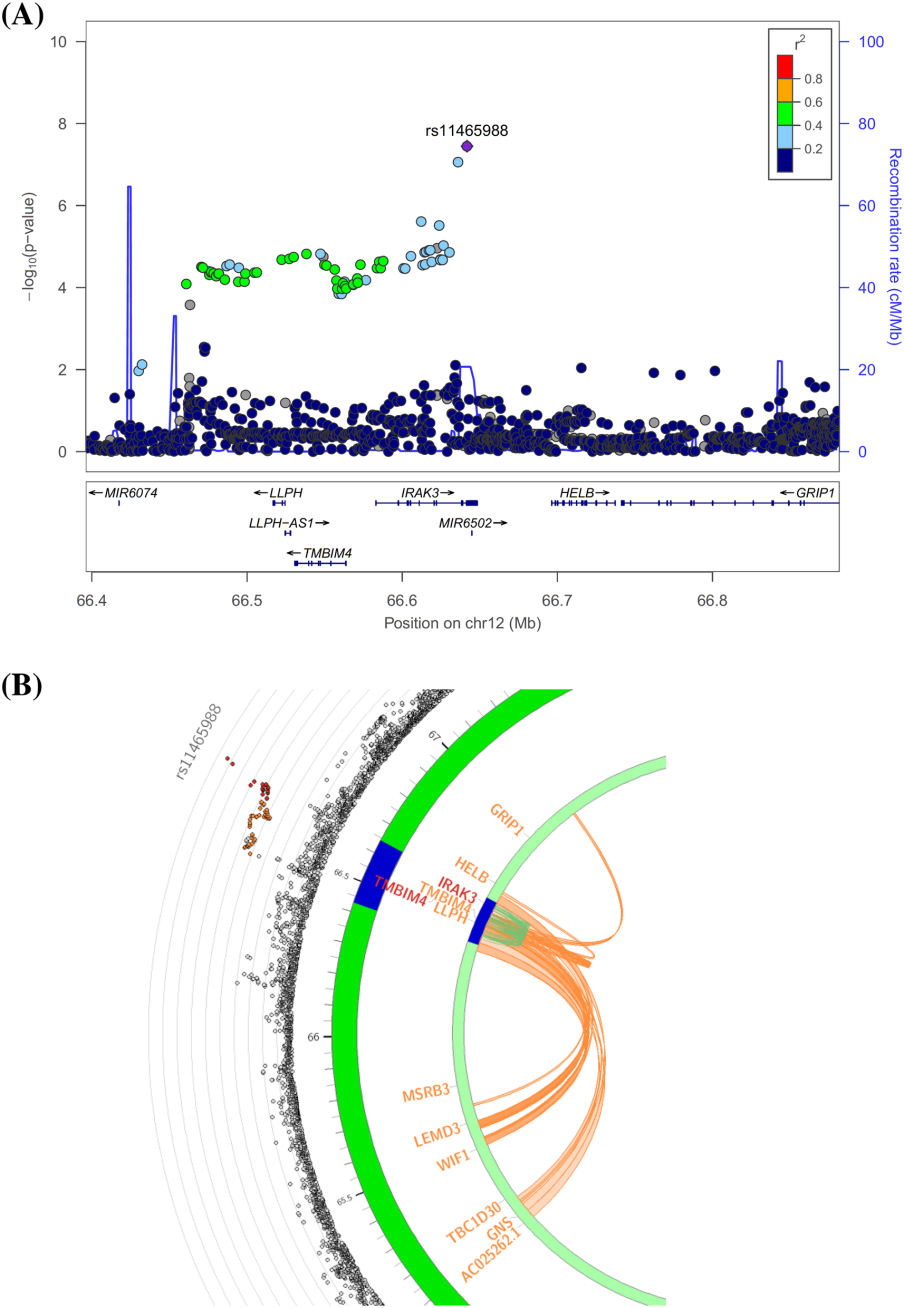
Genome-wide significant locus *IRAK3*. (**A**) Regional association plot; (**B**) circos plot. For the regional association plot generated via LocusZoom^52^ v1.4 (https://locuszoom.sph.umich.edu/), SNPs in genomic risk loci are color-coded as a function of their r^2^ to the index SNP rs11465988 in the locus, while SNPs with missing LD information are shown in grey. For the circos plot generated via FUMA^51^ v1.3.5e (https://fuma.ctglab.nl/), the outer most layer is Manhattan plot and the middle layer highlights genomic risk loci (as defined by FUMA^51^ using minimum P-value of lead SNPs of 1 × 10^–5^ and default values for other parameters) in blue, while the inner most layer highlights eQTLs and/or chromatin interactions. Only SNPs with *p* < 0.05 are displayed in the outer ring. SNPs in genomic risk loci are color-coded as a function of their maximum r^2^ to the one of the independent significant SNPs in the locus. The rsID of the top SNPs in each risk locus are displayed in the most outer layer. For the inner most layer, if the gene is mapped only by chromatin interactions or only by eQTLs, it is colored orange or green, respectively. It is colored red when the gene is mapped by both.

The genome-wide association analysis revealed one genome-wide significant association between an exonic synonymous variant (rs11465988, *p* = 3.57 × 10^–8^) in the interleukin 1 receptor associated kinase 3 (*IRAK3*) gene and the percent change in MADRS score (Table 2, Fig. 1 for Manhattan plot, Fig. 2A for regional plot and Supplemental Fig. 1A for QQ plot, Genomic Control lambda (λ) = 0.986). SNPs (e.g. rs115989442, rs144324167, rs79138866, rs116371327, rs150373274, and rs144520864) in linkage disequilibrium (r^2^ = 0.64) with rs11465988 are part of the regions engaging in intra-chromosomal loop (Fig. 2B, Supplemental Table 2) and could potentially be regulatory elements. rs144520864 is in fact located in a region with an annotated enhancer. An additional regional plot using rs17767394 as index SNP is also shown as Supplemental Fig. 2.

**Table 2:** Variants with association p-value less than 1 × 10^–6^ in GWAS.

rsID	Chr	pos	A1	A2	FRQ	INFO	Beta/OR	SE	p	Func.refGene	Gene.refGene	GeneDetail.refGene
Percentage change of MADRS from baseline												
rs11465988	12	66641813	C	T	0.9898	0.51	− 51.6	9.2	3.57E−08	Exonic	IRAK3	
rs17767394	12	66636086	C	A	0.9843	0.78	− 32.7	6	8.68E−08	Intronic	IRAK3	
rs4739050	8	64034747	G	A	0.3376	1.02	7.5	1.4	6.06E−08	Intergenic	TTPA;YTHDF3-AS1	dist = 36135; dist = 45537
rs151184257	4	105714757	A	G	0.9888	0.61	− 40.5	7.9	4.51E−07	Intergenic	CXXC4-AS1;TET2	dist = 96008; dist = 352275
rs115141868	2	70816605	A	C	0.9898	0.48	− 46.8	9.3	7.65E−07	Intergenic	TGFA;ADD2	dist = 35458; dist = 72611
Response status												
rs10957273	8	6.4E+07	T	C	0.3028	1	0.3	0.2	8.07E−07	Intergenic	TTPA;YTHDF3-AS1	dist = 30479; dist = 51193

Note that beta coefficient is reported for percentage change of MADRS score from baseline and OR is reported for responder status.

The other two GWAS for responder and remission status, respectively, did not yield any genome-wide significant finding (Supplemental Figs. 3A,B for Manhattan plots; Supplemental Figs. 1B,C for QQ plots, λ = 1.028 and 0.997, respectively). Nevertheless, a suggestive signal that merits comment was identified in chromosome 8 (rs4739050, nominal *p* = 6.06 × 10^–8^, β = 7.5, SE = 1.4 for percentage change in MADRS score; rs10957273, nominal *p* = 8.07 × 10^–7^, OR = 0.3, SE = 0.2 for responder status) from both the continuous endpoint GWAS and the responder status GWAS. Rs4739050 is an expression quantitative trait locus (eQTL) for gamma-glutamyl hydrolase (*GGH*) based on eQTLGen (*p*_*eQTL*_ = 4.24 × 10^–9^). A full list of suggestive associations with p-values less than 1 × 10^–4^ is provided in Supplemental Table 3.

Gene-level association analysis revealed one significant gene NME/NM23 family member 7 (*NME7, p* = 1.73 × 10^–6^, Supplemental Fig. 4A) for the percentage change in MADRS score. In the percent change in MADRS score GWAS, a pathway enrichment analysis revealed suggestive enrichments of genes involved in the negative regulation of glucocorticoid metabolic process (nominal *p* = 3.53 × 10^–5^) and neuronal action potential (nominal *p* = 0.0001). Pathway enrichment analysis also revealed suggestive (p-values listed are nominal) enrichments of genes involved in synaptic vesicle clustering (*p* = 4.33 × 10^–5^), negative regulation of glucocorticoid metabolic process (*p* = 5.48 × 10^–5^), regulation of synaptic vesicle clustering (*p* = 6.13 × 10^–5^), anterior posterior axon guidance (*p* = 0.0002), and netrin mediated repulsion signals (*p* = 0.0002) in the responder status GWAS, and in the negative regulation of extrinsic apoptotic signaling pathway (*p* = 4.04 × 10^–5^), NF-κB canonical pathway (*p* = 5.90 × 10^–5^), stress pathway (*p* = 0.0002), and TNFR1 induced proapoptotic signaling (*p* = 0.0003) in the remission status GWAS (Supplemental Table 4). We did not identify an association between the change in MADRS score and the *BDNF* Val66Met polymorphism in the current study (*p* > 0.05).

After applying corrections for multiple testing, none of the associations between esketamine’s antidepressant efficacy and the PRS genetic loading for psychiatric conditions or symptom profiles was significant at the study-wide level (which required *p* < 0.0004 for significance). In Table 3 and Supplemental Fig. 5 we list suggestive associations observed in these analyses, however, along with their nominal p-values. Thus we observed suggestive (i.e., p-values listed are nominal) negative correlations between the depressive symptom PRS^15^ (*p* = 0.001, Table 3 and Supplemental Fig. 5) and the esketamine treatment response outcome as measured by percentage change from baseline in the MADRS score at the end of four week treatment period (Table 3). In addition, the depressive symptom PRS (*p* = 0.004) displayed suggestive positive correlations with esketamine responder status. Lastly, depressive symptoms PRS (*p* = 0.002) and insomnia^16^ PRS (*p* = 0.003) exhibited suggestive positive correlations with esketamine remission status.

**Table 3:** Polygenic Risk Score association with esketamine treatment outcome.

Threshold	r^2^_PRS_	r^2^_Full_	r^2^_Null_	Standardized coefficient	Standard error	p	Number of SNP	Base GWAS	References
**Percentage change of MADRS from baseline**									
0.05	0.017351	0.152098	0.134747	− 3.06	0.94	1.20E−03	13,443	Depressive symptoms	Okbay et al., 2016
0.001	0.0111608	0.145908	0.134747	− 2.50	0.96	9.54E−03	1,000	ADHD	Demontis et al., 2019
0.001	0.00761273	0.14236	0.134747	2.02	0.94	3.25E−02	245	Anxiety	Otowa et al., 2016
0.001	0.00699865	0.141746	0.134747	2.04	0.99	4.03E−02	2,742	PGC2_SCZ	Ripke et al., 2014
0.3	0.00638174	0.141129	0.134747	− 1.95	0.99	5.03E−02	44,314	Insomnia	Hammerschlag et al., 2017
0.001	0.00521661	0.139964	0.134747	1.82	1.03	7.69E−02	1,362	PGC2_BIP	Stahl et al., 2019
0.001	0.00354911	0.138297	0.134747	− 1.42	0.97	1.45E−01	1,917	PGC2_MDD+UKB	Howard et al., 2019
0.5	0.00295958	0.137707	0.134747	− 3.77	2.83	1.83E−01	60,258	SA_in_MDD_BIP_SCZ	Mullins et al., 2019
0.001	0.00229691	0.137044	0.134747	− 1.36	1.16	2.41E−01	471	SA_in_MDD	Mullins et al., 2019
0.001	0.00101084	0.135758	0.134747	0.76	0.98	4.37E−01	5,295	EA	Lee et al., 2018
0.05	0.000785382	0.135533	0.134747	− 0.72	1.06	4.93E−01	12,344	ASD	Grove et al., 2019
0.05	0.000728554	0.135476	0.134747	0.65	0.98	5.09E−01	10,729	SWB	Okbay et al., 2016
0.05	0.000634123	0.135382	0.134747	− 0.58	0.95	5.38E−01	18,460	CP	Lee et al., 2018
0.05	0.0006288	0.135376	0.134747	− 0.59	0.97	5.40E−01	13,743	Neuroticism	Okbay et al., 2016
0.001	0.00050388	0.135251	0.134747	− 0.53	0.96	5.83E−01	6,764	BMI	Yengo et al., 2018
**Response status**									
0.05	0.0218424	0.34341	0.321568	0.43	0.15	4.39E−03	13,443	Depressive symptoms	Okbay et al., 2016
1	0.018916	0.340484	0.321568	0.41	0.15	7.83E−03	77,733	ADHD	Demontis et al., 2019
0.4	0.0182367	0.339804	0.321568	− 0.64	0.25	9.57E−03	56,270	PGC2_SCZ	Ripke et al., 2014
0.001	0.0103815	0.331949	0.321568	− 0.28	0.14	5.02E−02	245	Anxiety	Otowa et al., 2016
0.5	0.00654157	0.328109	0.321568	0.69	0.44	1.16E−01	60,258	SA_in_MDD_BIP_SCZ	Mullins et al., 2019
0.2	0.0050513	0.326619	0.321568	0.20	0.15	1.67E−01	34,449	Insomnia	Hammerschlag et al., 2017
0.001	0.00454651	0.326114	0.321568	− 0.19	0.14	1.89E−01	5,295	EA	Lee et al., 2018
0.05	0.0041483	0.325716	0.321568	− 0.19	0.15	2.10E−01	10,729	SWB	Okbay et al., 2016
0.001	0.00366392	0.325232	0.321568	− 0.16	0.14	2.39E−01	733	ASD	Grove et al., 2019
0.4	0.00251489	0.324083	0.321568	0.34	0.35	3.30E−01	52,541	SA_in_MDD	Mullins et al., 2019
0.1	0.00228732	0.323855	0.321568	− 0.15	0.16	3.53E−01	24,185	PGC2_MDD+UKB	Howard et al., 2019
0.001	0.00184734	0.323415	0.321568	0.12	0.14	4.02E−01	6,764	BMI	Yengo et al., 2018
0.05	0.000794085	0.322362	0.321568	− 0.13	0.24	5.83E−01	15,002	PGC2_BIP	Stahl et al., 2019
0.3	0.000469917	0.322038	0.321568	− 0.06	0.15	6.73E−01	47,523	CP	Lee et al., 2018
0.001	0.000213117	0.321781	0.321568	− 0.04	0.14	7.76E−01	1,108	Neuroticism	Okbay et al., 2016
**Remission status**									
0.05	0.020447	0.218933	0.198486	0.30	0.10	2.29E−03	13,443	Depressive symptoms	Okbay et al., 2016
1	0.019018	0.217504	0.198486	0.31	0.10	3.25E−03	79,083	Insomnia	Hammerschlag et al., 2017
0.001	0.0131928	0.211679	0.198486	− 0.26	0.11	1.41E−02	1,362	PGC2_BIP	Stahl et al., 2019
0.001	0.0127337	0.211219	0.198486	− 0.25	0.10	1.61E−02	2,742	PGC2_SCZ	Ripke et al., 2014
0.001	0.0103469	0.208833	0.198486	0.22	0.10	2.93E−02	1,917	PGC2_MDD+UKB	Howard et al., 2019
0.05	0.00709676	0.205582	0.198486	− 0.18	0.10	7.04E−02	6,160	Anxiety	Otowa et al., 2016
0.1	0.00668848	0.205174	0.198486	− 0.18	0.10	7.90E−02	20,193	ASD	Grove et al., 2019
0.5	0.00611421	0.2046	0.198486	0.48	0.29	9.31E−02	60,258	SA_in_MDD_BIP_SCZ	Mullins et al., 2019
0.001	0.00482744	0.203313	0.198486	0.15	0.10	1.35E−01	1,000	ADHD	Demontis et al.,2019
0.05	0.00292355	0.201409	0.198486	0.11	0.10	2.45E−01	13,743	Neuroticism	Okbay et al., 2016
0.5	0.00220535	0.200691	0.198486	0.10	0.10	3.12E−01	61,486	CP	Lee et al., 2018
0.001	0.00119781	0.199684	0.198486	− 0.07	0.10	4.56E−01	5,295	EA	Lee et al., 2018
0.001	0.00113909	0.199625	0.198486	0.09	0.12	4.67E−01	471	SA_in_MDD	Mullins et al., 2019
1	0.00091008	0.199396	0.198486	0.07	0.10	5.16E−01	67,436	SWB	Okbay et al., 2016
0.3	0.000424952	0.198911	0.198486	− 0.04	0.10	6.57E−01	40,520	BMI	Yengo et al.,2018

*MDD* major depressive disorder, *BIP* bipolar disorder, *SCZ* schizophrenia, *ADHD* attention deficit/hyperactivity disorder, *ASD* autism, *SWB* subjective well-being, *CP* cognitive performance, *EA* education attainment, *UKB* UK Biobank, *PGC* Psychiatric Genomic Consortium.

*For the reported standardized coefficient in this table, only PRS was scaled while the dependent variable was kept in its original scale.

## Discussion

In this investigation of genetic associations with the antidepressant outcome to esketamine treatment, two findings remained significant after applying corrections for multiple testing. From the GWAS, a genome-wide significant association was identified with the percent change in MADRS score in an exonic SNP in *IRAK3*. *IRAK3* encodes a member of the interleukin-1 receptor-associated kinase protein family that is primarily expressed in monocytes and macrophages, where it functions as a negative regulator of Toll-like receptor signaling. In addition, the gene-level association analysis revealed one significant gene *NME7* for the percentage change in MADRS score. NME7 is a γ-tubulin ring complex component that regulates the microtubule-nucleating activity of this complex^17^.

A suggestive signal observed in both the continuous endpoint GWAS and the responder status GWAS was an expression quantitative trait loci (eQTL) for *GGH*, an enzyme that regulates intracellular folate concentrations. Folate deficiency has been linked to oxidative stress^18^. Meta-analysis showed that individuals with depression had lower folate levels than those without depression^19^. Folic acid administration was also shown to ameliorate depression-like behavior in rats subjected to chronic unpredictable mild stress, a putative rodent depression model^20^.

The previously reported association between the Val66Met *BDNF* variant and antidepressant response to IV ketamine was not replicated in the current study. A significant methodological difference between studies, however, was that in the previous trial that reported this association the antidepressant response was assessed 4 h post ketamine infusion^12^, whereas in the current study, the antidepressant outcome was assessed after 4 weeks of repeated esketamine nasal spray administration. While the importance of this timing difference in detecting an association with the Val66Met *BDNF* variant remains unclear, it is noteworthy that hypotheses generated from relatively small candidate gene studies in MDD have typically proven difficult to replicate in larger samples^14^, emphasizing the importance of studying larger sample sizes to detect reliable genetic signals. Nevertheless, the findings from the current study also warrant replication in larger sample sizes given the relatively modest number of participants included.

It has been shown previously that apoptotic biochemical cascades can exert local actions on the functions and structural dynamics of growth cones and synapses^21^. In this context, it is of interest that several apoptotic signaling pathways were identified as suggestive enriched gene sets. In preclinical models ketamine enhances structural plasticity in mouse mesencephalic and human iPSC-derived dopaminergic neurons via AMPAR-driven BDNF and rapamycin kinase (mTOR) signaling^22^. In a rat traumatic brain injury (TBI) model, posttraumatic administration of a sub-anesthetic dose of ketamine exerts neuroprotection via attenuating inflammation and autophagy^23^. There have been conflicting reports as to whether ketamine induces apoptosis, which might reflect a dependence on dose and developmental period. It was reported that ketamine induced apoptosis in human uroepithelial SV-HUC-1 cells^24^ and in the neonatal rat brain^25^, while a study in chronic unpredictable stress model of depression suggested an anti-apoptotic and antidepressant effects of ketamine^26^. In the exposure range encompassing concentrations at which esketamine nasal spray has been tested for antidepressant effects in humans, no evidence of neuronal toxicity was identified in experimental animals^27^. Notably, in a putative rodent depression model involving chronic mild stress that produces dendritic atrophy in the medial prefrontal cortex, a single ketamine administration restored synaptic density and function toward normative levels^28^. Such changes in synaptic plasticity are hypothesized to underlie the relatively long-lasting antidepressant effects of ketamine and esketamine following single or pulsed intermittent doses^29^, and the pathophysiology of MDD is associated with regional atrophy in the medial prefrontal cortex and other anatomically related structures^30^. In clinical studies the antidepressant response to ketamine has been predicted by peripheral blood evidence of low-grade inflammation at baseline or by the enhancement of stimulus-evoked somatosensory cortical responses (a putative in vivo measure of long term potentiation effects mediated via changes in synaptic plasticity) at 4 h post-administration^31,32^. Pathways relevant to neuronal and synaptic function, immune signaling, and glucocorticoid receptor/stress response showed enrichment among the GWAS suggestive signals. These findings are consistent with the hypotheses that inflammation and synaptic plasticity play a role in differentiating esketamine responders from non-responders.

This study suggests that PRS for psychopathology/symptom profiles may influence the antidepressant treatment outcome for esketamine. Although the PRS for depressive symptoms^15^ did not reach study-wide significance, the suggestive associations were consistent across multiple p-value thresholds (*p*_T_) used to construct PRS and across three esketamine efficacy endpoints. In addition, the condition of “depression”^33^ PRS (PGC2_MDD + UKB in Table 3) constructed by the summary statistics from the GWAS meta-analysis by Howard et al. (2019) (without the 23andMe cohort^34^) showed suggestive associations that did not reach significance for the remission endpoint. The GWAS meta-analyses for depressive symptoms^15^ and that for the condition, “depression”^33^ differ in two respects. First, while both studies included PGC phase 1 samples^35^, the GERA^36^ samples (7,231 cases and 49,316 controls), and the UK Biobank (UKB) samples, the “depression” GWAS meta-analysis^33^ additionally included samples from other cohorts, e.g. iPSYCH, deCODE, GenScot, and the incremental core samples from PGC MDD Working Group phase 2 analysis^36^. Second, the phenotypic definition differed in that ”depressive symptoms” in the UKB cohort from the Okbay et al. (2016) study employed a continuous phenotype measure by combining responses to two mental health questionnaire (MHQ) questions deployed to UKB participants, which asked about the frequency in the past two weeks with which the respondent experienced feelings of lack of enthusiasm/interest and depression/hopelessness, whereas the Howard et al. (2019) study used a “broad depression” phenotype^37^, e.g. self-reported past help-seeking for problems with “nerves, anxiety, tension or depression.

Finally, genetic loading of BMI was not associated with esketamine remission status, in contrast to the previous report of ketamine^10^. In a correlation analysis of the SUSTAIN-2 clinical data, baseline BMI was also not associated with remission status, either in the entire sample irrespective of race (*p* = 0.365, n = 667) or in the subsample with European ancestry (*p* = 0.742, Supplemental Table 1).

## Methods

PsychArray genotyping data were generated using blood DNA samples collected from the SUSTAIN-2^8^ (NCT02497287, n = 598) and TRANSFORM-3^2^ (NCT02422186, n = 95; only participants with age of onset less than 55 years were included) phase III pivotal clinical studies. All subjects genotyped were of European ancestry. The clinical studies were carried out in accordance with the ethical principles outlined in the Declaration of Helsinki, Good Clinical Practices guidelines, and applicable regulatory requirements. The study protocols were approved by the local, regional, or central Institutional Review Board (IRB) or Independent Ethics Committee (IEC) overseeing the respective clinical sites: Sterling Institutional Review Board, IRB—UConn Health, Human Research Protection Program (US); Comité de Etica e Investigación del Sanatorio Profesor, Comité de ética en Investigación de Winsett Rethman S.A. de C.V., Comité de Etica en Investigación del Hospital La Mision SA de CV (Mexico); Comité de Etica en Investigación (CEI-INAPSI), CEI Fundación Rusculleda, Comité de Etica en Investigacion Burzaco, Comité de Ética IPEM, Comité Institucional de Ética en Investigación en Salud CIEIS Hospital Italiano (Aregentina); Comite de Etica em Pesquisa da UNIFESP/EPM, Comite de Etica em Pesquisa do Complexo Hospitar HUOC/PROCAPE, Comite de Etica em Pesquisas do Hospital Pro-Cardiaco Rua Voluntarios da Patria (Brasil); Western Institutional Review Board, Oxford Health NHS Foundation Trust, Derbyshire Healthcare NHS Foundation Trust, South London and Maudsley NHS Foundation Trust, Northamptonshire Healthcare NHS Foundation Trust, Ashgate Medical Practice Ethics Committee, Oxfordshire Research Ethics Committee A (UK); Alfred Health Human Ethics Committee, Bellberry Limited (Australia); Regionala Etikprövningsnämnden i Lund, Komisja Bioetyczna przy Kujawsko-Pomorskiej OIL (Sweden); CPP ile de France VIII (France); Lithuanian Bioethics Committee (Lithuania); Comitato Etico per la sperimentazione clinica della Provincia di Vicenza (CESC-VI) (Italy); Naisten lasten ja psykiatrian eettinen toimikunta (Finland); Ethics Committee for Clinical Trials, AZ St.-Jan Brugge (Belgium); der Stadt Wien gemäß KAG, Ethik-Kommission der Landesärztekammer Brandenburg, Ethik-Kommission der Ärztekammer Westfalen-Lippe und der Medizinischen Fakultät der Westfälischen Wilhelms-Universität Münster (Germany); Uludag University Medical Faculty Clinical Research Ethics Committee, Dicle University Medical Faculty Clinical Research Ethical Committee (Turkey). All participants provided written informed consent before enrollment.

The analysis was composed of TRD patients who received esketamine combined with a newly initiated oral antidepressant treatment (SSRI or SNRI) either in an open labelled (for SUSTAIN-2) or in a randomized (for TRANSFORM-3) fashion. A total of 527 samples were included in the final analysis. Treatment response endpoints were defined as follow: (1) a quantitative trait using percentage of change of MADRS score at the end of study compared to baseline; (2) response defined as ≥ 50% improvement from baseline in the MADRS Score; (3) remission defined as MADRS ≤ 12 at study endpoint. Additional details of these clinical studies are provided in the Supplemental Text or could also be found in https://clinicaltrials.gov/.

Genotypes were imputed based on the 1000 Genome Project^38^ Phase I reference panel. A SNP-wise genome-wide association analysis was performed using PLINK^39,40^. In addition, a gene-wise genome-wide association followed by pathway enrichment analysis was performed using MAGMA^41^. In all analyses the models corrected for gender, study ID, baseline symptom severity, and 5 principal components representing the population substructure. Detailed methods are described in the Supplemental Text.

Polygenic risk scores (PRS) were constructed based on well-powered genome-wide association studies (GWAS) of 15 PRS phenotypes, of which six were constructed for psychiatric conditions (depression^33^, bipolar disorder^42^, schizophrenia^43^, autism^44^, ADHD^45^, anxiety^46^) and seven psychiatric characteristics (history of suicide attempt^47^ among depressive subjects or among schizophrenia, bipolar, and depressive subjects), depressive symptoms^15^, subjective well-being^15^, neuroticism^15^, insomnia^16^, education attainment^48^, and cognitive performance^48^), and BMI^49^. To correct the resulting p-values for performing comparisons in multiple PRS phenotypes and at 8 p-value thresholds assessed (i.e., 5e−08, 0.001, 0.05, 0.1,…0.5, 1), the association p-value < 0.05/(15 × 8) ~ 0.0004 (for 15 phenotypes and 8 P_T_ bins) between PRS and any esketamine treatment response outcome was considered to be study-wide significant. To balance Type 2 error, nonsignificant associations that reached nominal *p* < 0.005 were considered “suggestive”. The PRS analysis was performed using PRSice-2^50^. All p-values reported in this study were uncorrected p-values.

## Supplementary information

Supplementary Information 1.

Supplementary Information 2.

## References

[1] Singh JB (2016). Intravenous esketamine in adult treatment-resistant depression: a double-blind, double-randomization, placebo-controlled study. Biol. Psychiatry.

[2] Ochs-Ross R (2020). Efficacy and safety of esketamine nasal spray plus an oral antidepressant in elderly patients with treatment-resistant depression-TRANSFORM-3. Am. J. Geriatr. Psychiatry.

[3] Popova V (2019). Efficacy and safety of flexibly dosed esketamine nasal spray combined with a newly initiated oral antidepressant in treatment-resistant depression: a randomized double-blind active-controlled study. Am. J. Psychiatry.

[4] Fedgchin M (2019). Efficacy and safety of fixed-dose esketamine nasal spray combined with a new oral antidepressant in treatment-resistant depression: results of a randomized, double-blind, active-controlled study (TRANSFORM-1). Int. J. Neuropsychopharmacol..

[5] Daly EJ (2019). Efficacy of esketamine nasal spray plus oral antidepressant treatment for relapse prevention in patients with treatment-resistant depression: a randomized clinical trial. JAMA Psychiatry.

[6] Daly EJ (2018). Efficacy and safety of intranasal esketamine adjunctive to oral antidepressant therapy in treatment-resistant depression: a randomized clinical trial. JAMA Psychiatry.

[7] Canuso CM (2018). Efficacy and safety of intranasal esketamine for the rapid reduction of symptoms of depression and suicidality in patients at imminent risk for suicide: results of a double-blind, randomized, placebo-controlled study. Am. J. Psychiatry.

[8] Wajs E (2020). Esketamine nasal spray plus oral antidepressant in patients with treatment-resistant depression: assessment of long-term safety in a phase 3, open-label study (SUSTAIN-2). J. Clin. Psychiatry.

[9] Perlman K (2019). A systematic meta-review of predictors of antidepressant treatment outcome in major depressive disorder. J. Affect. Disord..

[10] Singh B (2019). The association between body mass index and remission rates in patients with treatment-resistant depression who received intravenous ketamine. J. Clin. Psychiatry.

[11] Liu RJ (2012). Brain-derived neurotrophic factor Val66Met allele impairs basal and ketamine-stimulated synaptogenesis in prefrontal cortex. Biol. Psychiatry.

[12] Laje G (2012). Brain-derived neurotrophic factor Val66Met polymorphism and antidepressant efficacy of ketamine in depressed patients. Biol. Psychiatry.

[13] Chen MH (2019). Antisuicidal effect, BDNF Val66Met polymorphism, and low-dose ketamine infusion: reanalysis of adjunctive ketamine study of Taiwanese patients with treatment-resistant depression (AKSTP-TRD). J. Affect. Disord..

[14] Border R (2019). No support for historical candidate gene or candidate gene-by-interaction hypotheses for major depression across multiple large samples. Am. J. Psychiatry.

[15] Okbay, A. *et al.* Genetic variants associated with subjective well-being, depressive symptoms, and neuroticism identified through genome-wide analyses. *Nat. Genet.* **48**, 624–633. 10.1038/ng.3552. https://www.nature.com/ng/journal/v48/n6/abs/ng.3552.html#supplementary-information (2016). 10.1038/ng.3552 PMC4884152 27089181

[16] Hammerschlag, A. R. *et al.* Genome-wide association analysis of insomnia complaints identifies risk genes and genetic overlap with psychiatric and metabolic traits. *Nat. Genet.* **49**, 1584. 10.1038/ng.3888. https://www.nature.com/articles/ng.3888#supplementary-information (2017). 10.1038/ng.3888 PMC5600256 28604731

[17] Liu P, Choi YK, Qi RZ (2014). NME7 is a functional component of the gamma-tubulin ring complex. Mol. Biol. Cell..

[18] Kao TT (2014). Folate deficiency-induced oxidative stress contributes to neuropathy in young and aged zebrafish–implication in neural tube defects and Alzheimer's diseases. Neurobiol. Dis..

[19] Bender A, Hagan KE, Kingston N (2017). The association of folate and depression: a meta-analysis. J. Psychiatr. Res..

[20] Zou F (2012). Brain expression genome-wide association study (eGWAS) identifies human disease-associated variants. PLoS Genet..

[21] Gilman CP, Mattson MP (2002). Do apoptotic mechanisms regulate synaptic plasticity and growth-cone motility?. Neuromol. Med..

[22] Cavalleri L (2018). Ketamine enhances structural plasticity in mouse mesencephalic and human iPSC-derived dopaminergic neurons via AMPAR-driven BDNF and mTOR signaling. Mol. Psychiatry.

[23] Wang CQ (2017). Posttraumatic administration of a sub-anesthetic dose of ketamine exerts neuroprotection via attenuating inflammation and autophagy. Neuroscience.

[24] Huang L (2014). Ketamine induces apoptosis of human uroepithelial SV-HUC-1 cells. Zhong nan da xue xue bao Yi xue ban.

[25] Soriano SG (2010). Ketamine activates cell cycle signaling and apoptosis in the neonatal rat brain. Anesthesiology.

[26] Liu WX (2016). Regulation of glutamate transporter 1 via BDNF-TrkB signaling plays a role in the anti-apoptotic and antidepressant effects of ketamine in chronic unpredictable stress model of depression. Psychopharmacology.

[27] Food and Drug Administration. Esketamine clinical review. (2019).

[28] Li N (2011). Glutamate *N*-methyl-d-aspartate receptor antagonists rapidly reverse behavioral and synaptic deficits caused by chronic stress exposure. Biol. Psychiatry.

[29] Duman RS, Aghajanian GK, Sanacora G, Krystal JH (2016). Synaptic plasticity and depression: new insights from stress and rapid-acting antidepressants. Nat. Med..

[30] Price JL, Drevets WC (2012). Neural circuits underlying the pathophysiology of mood disorders. Trends Cogn. Sci..

[31] Yang JJ (2015). Serum interleukin-6 is a predictive biomarker for ketamine's antidepressant effect in treatment-resistant patients with major depression. Biol. Psychiatry.

[32] Cornwell BR (2012). Synaptic potentiation is critical for rapid antidepressant response to ketamine in treatment-resistant major depression. Biol. Psychiatry.

[33] Howard DM (2019). Genome-wide meta-analysis of depression identifies 102 independent variants and highlights the importance of the prefrontal brain regions. Nat. Neurosci..

[34] Hyde CL (2016). Identification of 15 genetic loci associated with risk of major depression in individuals of European descent. Nat. Genet..

[35] Major Depressive Disorder Working Group of the Psychiatric GWAS Consortium (2013). A mega-analysis of genome-wide association studies for major depressive disorder. Mol. Psychiatry.

[36] Wray NR (2018). Genome-wide association analyses identify 44 risk variants and refine the genetic architecture of major depression. Nat. Genet..

[37] Howard DM (2018). Genome-wide association study of depression phenotypes in UK Biobank identifies variants in excitatory synaptic pathways. Nat. Commun..

[38] Genomes Project (2010). A map of human genome variation from population-scale sequencing. Nature.

[39] Purcell S (2007). PLINK: a tool set for whole-genome association and population-based linkage analyses. Am. J. Hum. Genet..

[40] Chang CC (2015). Second-generation PLINK: rising to the challenge of larger and richer datasets. Gigascience.

[41] de Leeuw CA, Mooij JM, Heskes T, Posthuma D (2015). MAGMA: generalized gene-set analysis of GWAS data. PLoS Comput. Biol..

[42] Stahl EA (2019). Genome-wide association study identifies 30 loci associated with bipolar disorder. Nat. Genet..

[43] Schizophrenia Working Group of the Psychiatric Genomics Consortium (2014). Biological insights from 108 schizophrenia-associated genetic loci. Nature.

[44] Grove J (2019). Identification of common genetic risk variants for autism spectrum disorder. Nat. Genet..

[45] Demontis D (2019). Discovery of the first genome-wide significant risk loci for attention deficit/hyperactivity disorder. Nat. Genet..

[46] Otowa T (2016). Meta-analysis of genome-wide association studies of anxiety disorders. Mol. Psychiatry.

[47] Mullins N (2019). GWAS of suicide attempt in psychiatric disorders and association with major depression polygenic risk scores. Am. J. Psychiatry.

[48] Lee JJ (2018). Gene discovery and polygenic prediction from a genome-wide association study of educational attainment in 1.1 million individuals. Nat. Genet..

[49] Yengo L (2018). Meta-analysis of genome-wide association studies for height and body mass index in approximately 700000 individuals of European ancestry. Hum. Mol. Genet..

[50] Choi SW, O'Reilly PF (2019). PRSice-2: Polygenic Risk Score software for biobank-scale data. Gigascience.

[51] Watanabe K, Taskesen E, van Bochoven A, Posthuma D (2017). Functional mapping and annotation of genetic associations with FUMA. Nat. Commun..

[52] Pruim RJ (2010). LocusZoom: regional visualization of genome-wide association scan results. Bioinformatics.

