# Supplementary material for: Genome-wide association study and polygenic risk score analysis of esketamine treatment response
Source: Sci Rep. 2020 Jul 28;10:12649. doi: 10.1038/s41598-020-69291-6 (PMC7387452; doi:10.1038/s41598-020-69291-6)
Supplement: Supplementary file 1 — Supplementary Information 1. [file 41598_2020_69291_MOESM1_ESM.docx]

**GENOME-WIDE ASSOCIATION STUDY AND POLYGENIC RISK SCORE ANALYSIS OF ESKETAMINE TREATMENT RESPONSE**

Qingqin S. Li^1^, Ewa Wajs^2^, Rachel Ochs-Ross^1^, Jaskaran Singh^3,a^, Wayne C. Drevets^3^

^1^Neuroscience Therapeutic Area, Janssen Research & Development, LLC, 1125 Trenton-Harbourton Road, Titusville, NJ; ^3^Neuroscience Therapeutic Area, Janssen Research & Development, LLC, Beerse, Belgium; ^3^Neuroscience Therapeutic Area, Janssen Research & Development, LLC, La Jolla, CA

Correspondence: Qingqin S Li, Ph.D. (qli2@its.jnj.com)

**^a^Current address:** Neurocrine Biosciences, Inc., 12780 El Camino Real, San Diego, CA 92130

**Supplemental Material**

**Supplemental Text**

**Supplemental Table 1** Characteristics of study participants by study

**Supplemental Table 2** A list of regions from *IRAK3* region and studies implicated in intra-chromatin interactions

**Supplemental Table 3** A list of suggestive association with p-value less than 1 x 10^-4^ (Please see the separate Excel spreadsheet)

**Supplemental Table 4** MAGMA gene set enrichment analysis

**Supplemental Figure 1** QQ plots of GWAS

**Supplemental Figure 2** Regional plot using rs17767394 as index SNP

**Supplemental Figure 3** Manhattan plots of SNP-based GWAS

**Supplemental Figure 4** Manhattan plots of gene-based association test

**Supplemental Figure 5** Suggestive association of depression symptom PRS with esketamine treatment response outcome across different p-value threshold (P_T)_ in base GWAS

**Supplemental Figure 6** Population substructure for the subjects retained in the GWAS analysis

**Supplemental Text**

**Methods**

**Clinical cohorts**

SUSTAIN-2 (NCT02497287) was an open-label multicenter, long-term study to evaluate the safety and efficacy of intranasal esketamine plus a newly initiated oral antidepressant in subjects with TRD.[^1^](#_ENREF_1) Subjects entered the study either directly (referred to as ‘direct-entry subjects’) or after completing the

double-blind induction phase of TRANSFORM-3[^2^](#_ENREF_2) (NCT02422186), a short-term efficacy study, in elderly

subjects with TRD (referred to as ‘transferred-entry subjects’). Approximately 750 direct entry

subjects were planned to be enrolled in this study, plus transferred-entry subjects from study

TRANSFORM-3. TRANSFORM-3 was a randomized, double-blind, active-controlled, 4-week study in male and female elderly subjects (>= 65 years) with TRD to assess the efficacy, safety, and tolerability of

flexibly dosed intranasal esketamine (28 mg, 56 mg, or 84 mg) plus a newly initiated oral

antidepressant, compared with a newly initiated oral antidepressant (active comparator) plus

intranasal placebo.

Each subject was required to meet DSM-5 diagnostic criteria of single-episode MDD (if single episode MDD, the duration must have been >= 2 years) or recurrent MDD, without psychotic features, based on clinical assessment and confirmed by the Mini International Neuropsychiatric Interview (MINI). At the start of the screening phase, each subject must have had non-response to >= 2 oral antidepressant treatments in the current episode of depression, as assessed using the MGH-ATRQ and confirmed by documented records (e.g., medical /pharmacy /prescription records or a letter from treating a physician, etc.). For specific tricyclic antidepressants which were being taken at a dose below the MGH-ATRQ minimum therapeutic dose, a blood level that is within the therapeutic (antidepressant) range, was acceptable to establish the adequacy of the antidepressant treatment. For SUSTAIN-2, each subject was required to have a MADRS total score of >= 22 at screening. The inclusion criteria for TRANSFORM-3 was similar to SUSTAIN-2 with the following exceptions: 1) At the time of signing the informed consent form (ICF), a subject must have been a man or woman 65 years of age or older; 2) a subject must have had nonresponse to >= 2 **but <= 5** oral antidepressant treatments in the current episode of depression, assessed using the MGH-ATRQ; 3) a subject must have been taking an oral antidepressant treatment with nonresponse at the start of the screening/prospective observational phase; 4) a subject must have been adherent to the continued oral antidepressant medication(s) (without adjustment in dosage) through the screening/prospective observational phase, as documented on the Patient Adherence Questionnaire (PAQ). Missing >= 4 days of antidepressant medication in the prior 2 week period will be considered as inadequate adherence; 5) a subject who was a non-responder to the antidepressant medication(s) from the screening/prospective observational phase (as assessed by independent, remote raters) was eligible for randomization if all other entry criteria were met; 6) a subject must have had an IDS-C_30_ total score of >= 34 at the start of the screening/prospective observational phase. In summary, the TRD status was determined retrospectively in SUSTAIN-2, and prospectively in TRANSFORM-3. The clinical data from the first 4 week period from the double blinded randomized treatment phase from TRANSFORM-3 or from the open labelled treatment SUSTAIN-2 trial were used in this pharmacogenomic study.

**Genotype data quality control**

The genetic samples were excluded if sample-wise missingness rate exceeded 1%, and genetic markers were excluded if SNP-wise missingness rate exceeded 5%, failed Hardy-Weinberg equilibrium (*p* < 1 x 10^-6^) or had minor allele frequency (MAF) below 1%. The total genotyping rate was 99.91%. Among the remaining participants, relatedness was assessed using pairwise identity by descent (IBD) estimation in PLINK.[^3^](#_ENREF_3) Participants were excluded as needed to ensure that the estimated proportion of IBD (PI_HAT) between any two remaining individuals was less than 0.3. Additionally, subjects with genetically inferred gender discrepancies from the gender provided in the clinical database were excluded. Four samples were excluded due to gender discrepancy check. Five rounds of outlier removal were applied to remove subjects 6 sigma away using EIGENSTRAT[^4^](#_ENREF_4)^,^[^5^](#_ENREF_5) (using default parameters except adding the options of nsnpldregress: 3 and maxdistldregress: 1) to strike a balance between keeping homogenous genetic subjects and loss of too many samples to preserve study power. Following these procedures, data on 527 subjects were retained for further analysis and the population substructure for these remaining subjects was shown in Supplemental Figure S6.

### Imputation

To allow the exploration of a greater density of markers than what was genotyped directly, we imputed genotypes using IMPUTE2 (v2.3.2).[^6-10^](#_ENREF_6) Imputation of unobserved genotypes was based on the reference haplotypes from the 1,000 Genomes haplotypes; Phase I integrated variant set release (SHAPEIT2) in NCBI build 37 (hg19) coordinates, singletons from the 1000 Genomes Project (1,092 individuals) were removed from autosomes. Internal cross validation was carried out automatically by IMPUTE2. The calculation was performed by masking one variant at a time in the study data, imputing the masked variant and comparing the result to the original genotype. Concordance for variants with maximum posterior probability > 90% (interval [0.9-1.0]) was > 96%.

### Genetic Association

~9M imputed and directly genotyped markers with imputation info score > 0.3 and MAF > 1% were used in the genome wide association analysis using PLINK. The imputed genotype dosages were assessed for association in a linear or logistic regression model, correcting for 5 principal components to account for population substructure. For the gene-based association test using MAGMA[^11^](#_ENREF_11), input SNPs were mapped to 18,760 protein coding genes. Genome wide significance was defined at *p* = 0.05/18,760 = 2.67 x 10^-6^ was considered as study-wide significant. The regional plot was created using LocusZoom (v1.4)[^12^](#_ENREF_12) available from <http://locuszoom.sph.umich.edu/>, while the Manhattan plots, QQ plots, and circos plots were generated using FUMA (v1.3.5e)[^13^](#_ENREF_13) available from <https://fuma.ctglab.nl/>.

**Table S1** Characteristics of study participants by study

|  | TRANSFORM-3 (n = 49) | SUSTAIN-2 (n = 478) |
| --- | --- | --- |
| Mean (%) |  |  |
| Age | 69.6 (4.5) | 50.2 (13.0) |
| Baseline BMI | 28.7 (5.0) | 28.2 (5.8) |
| Baseline MADRS score | 35.3 (6.0) | 31.0 (4.7) |
| Percentage change from baseline | -32.2 (32.9) | -58.3 (20.2) |
| N (%) |  |  |
| Race, White | 49 (100) | 478 (100) |
| Gender, Female | 31 (63.3) | 297 (62.1) |
| Remission status, remitted | 10 (20.4) | 245 (51.3) |
| Responder status, responder | 14 (28.6) | 429 (89.7) |
| Concomitant medications |  |  |
| DULOXETINE | 17 (34.7) | 160 (33.5) |
| ESCITALOPRAM | 21 (42.9) | 137 (28.7) |
| SERTRALINE | 7 (14.3) | 87 (18.2) |
| VENLAFAXINE XR | 4 (8.2) | 93 (19.5) |
| None |  | 1 (0.2) |

**Table S2** A list of regions from *IRAK3* region and studies implicated in intra-chromatin interactions

**Table S4** MAGMA gene set enrichment analysis

**Figure S1** QQ plots of GWAS (a) percentage change of MADRS score (Genomic Control lambda (λ) calculated based on the 50th percentile (median) = 0.986); (b) responder status (λ = 1.028); (c) remission status (λ = 0.997)

(a)


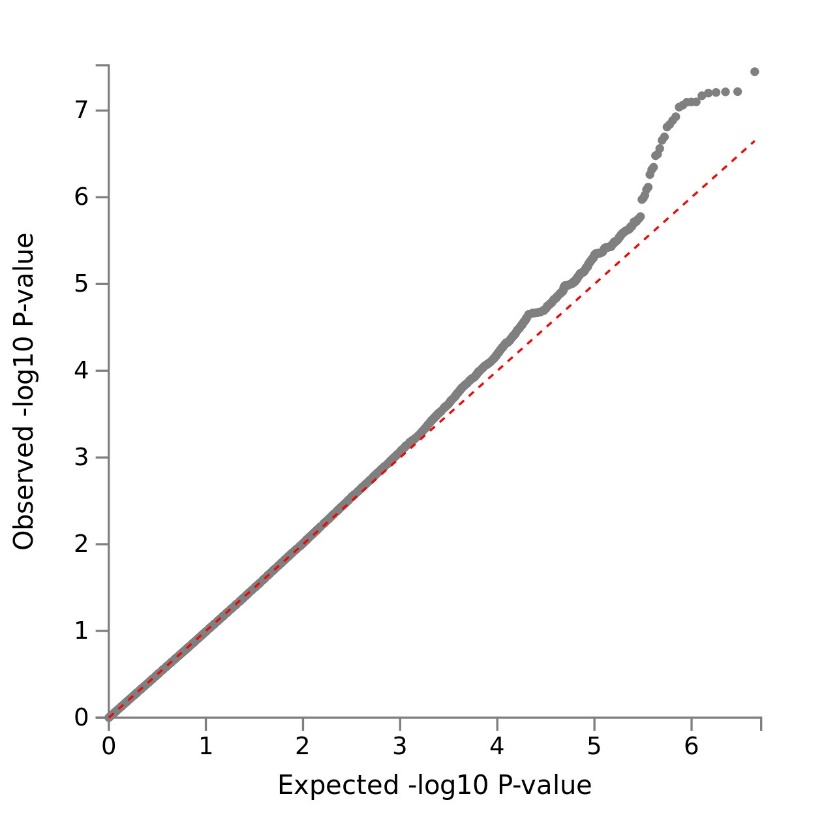


(b)


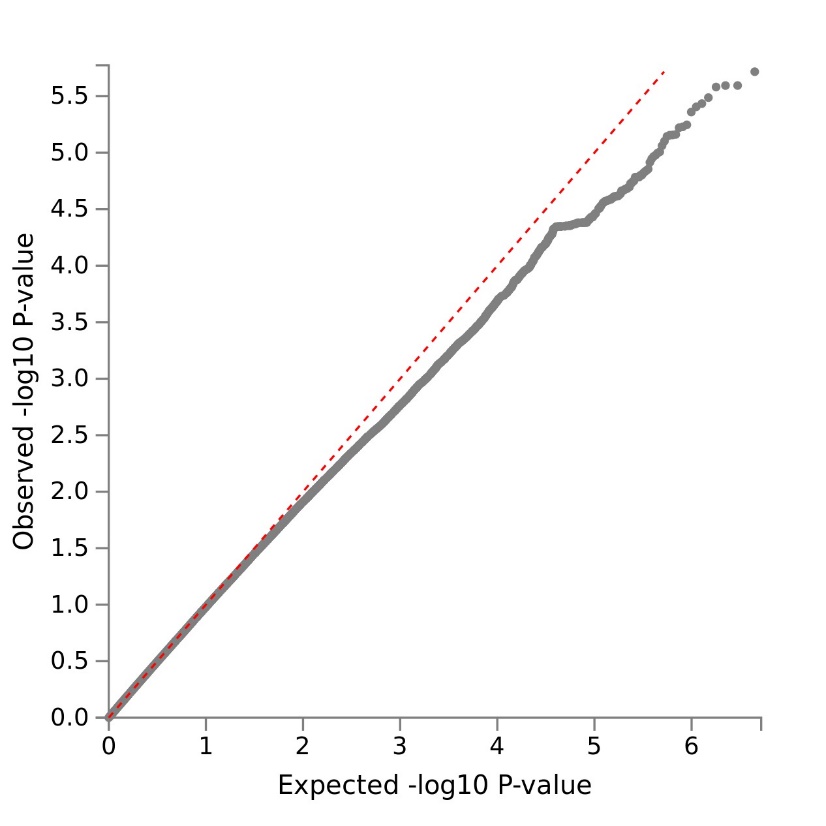


(c)

**
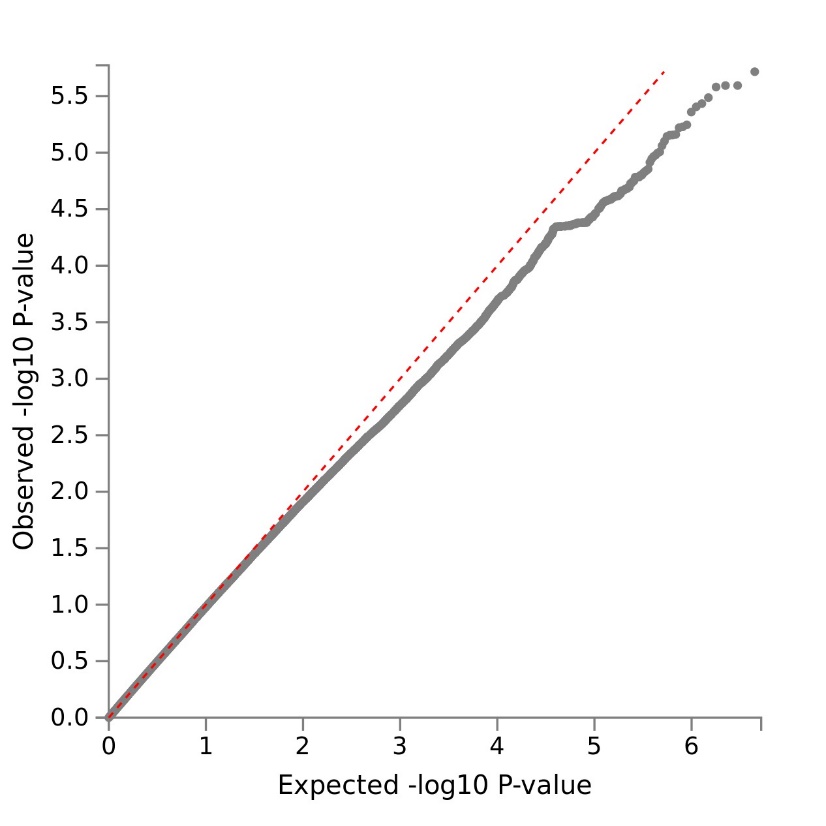
**

**Figure S2** Regional plot using rs17767394 as index SNP


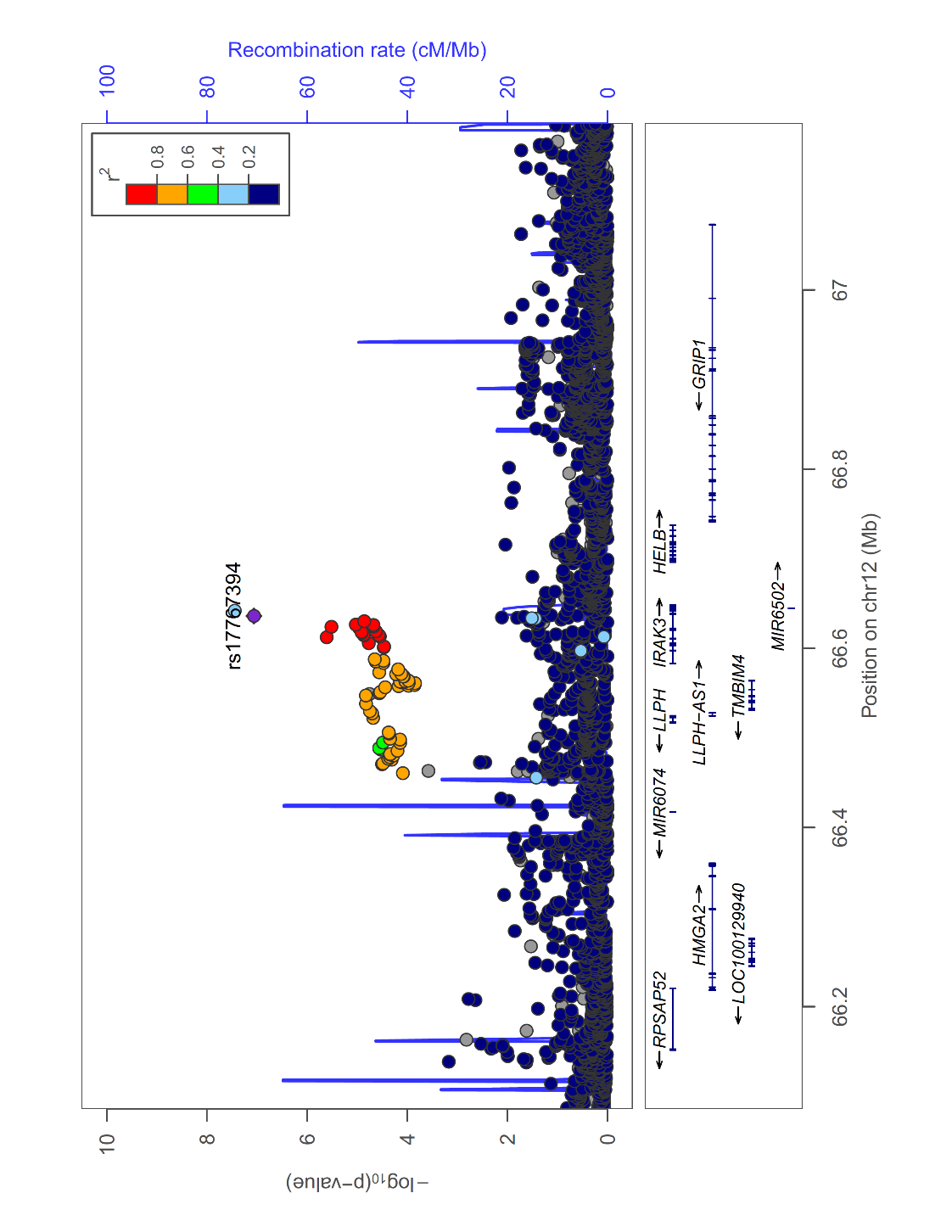


**Figure S3** Manhattan plots of SNP based GWAS (a) responder status; (b) remission status

(a)


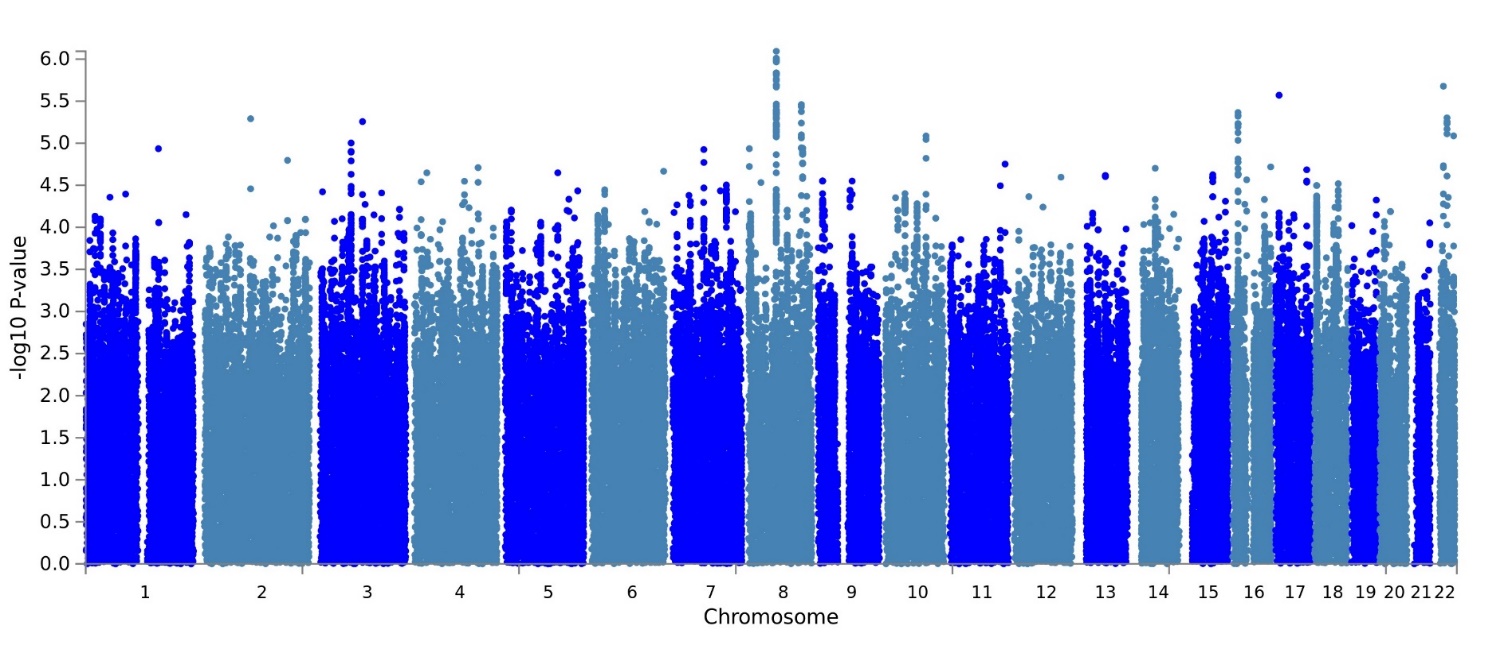


(b)


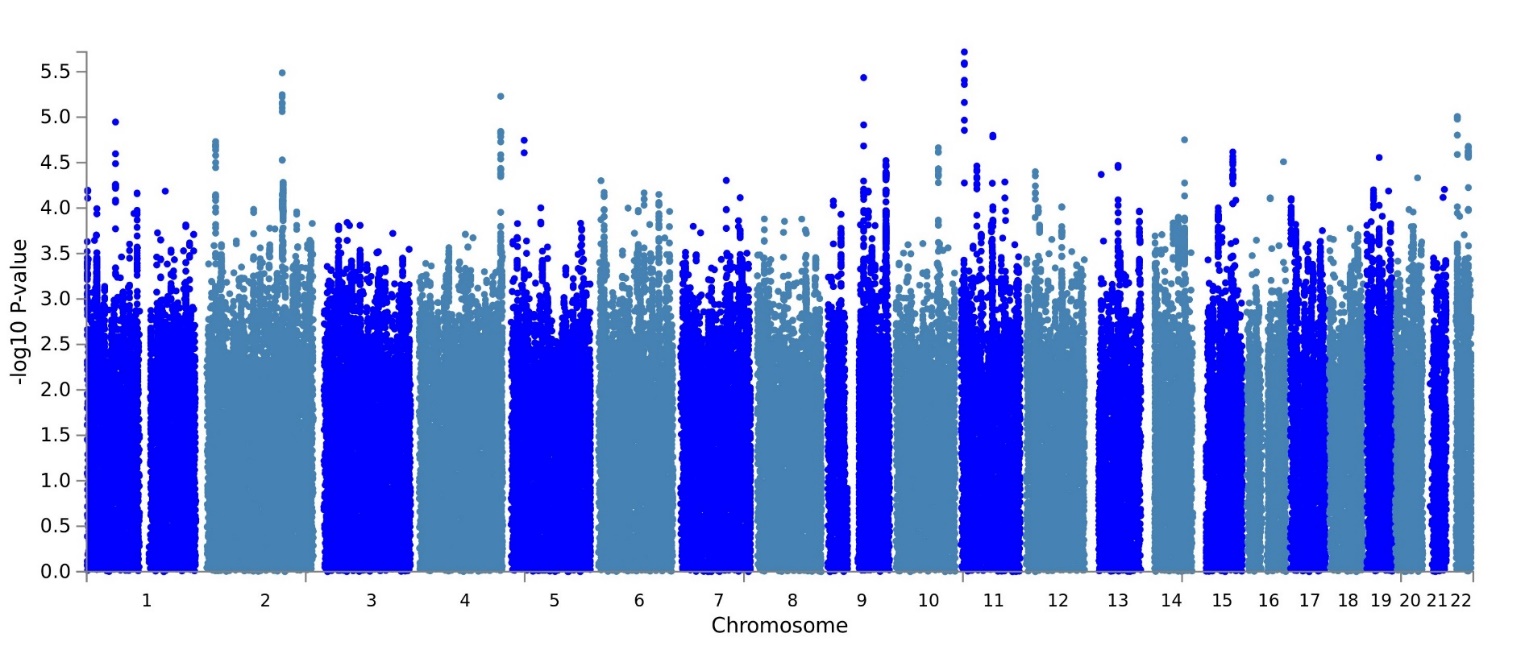


**Figure S4** Manhattan plots of gene-based association test (a) percentage change in MADRS; (b) responder status; (c) remission status. Red dashed line in the plot denotes genome wide significance threshold at p = 0.05/18760 = 2.67 x 10^-6^.

(a)


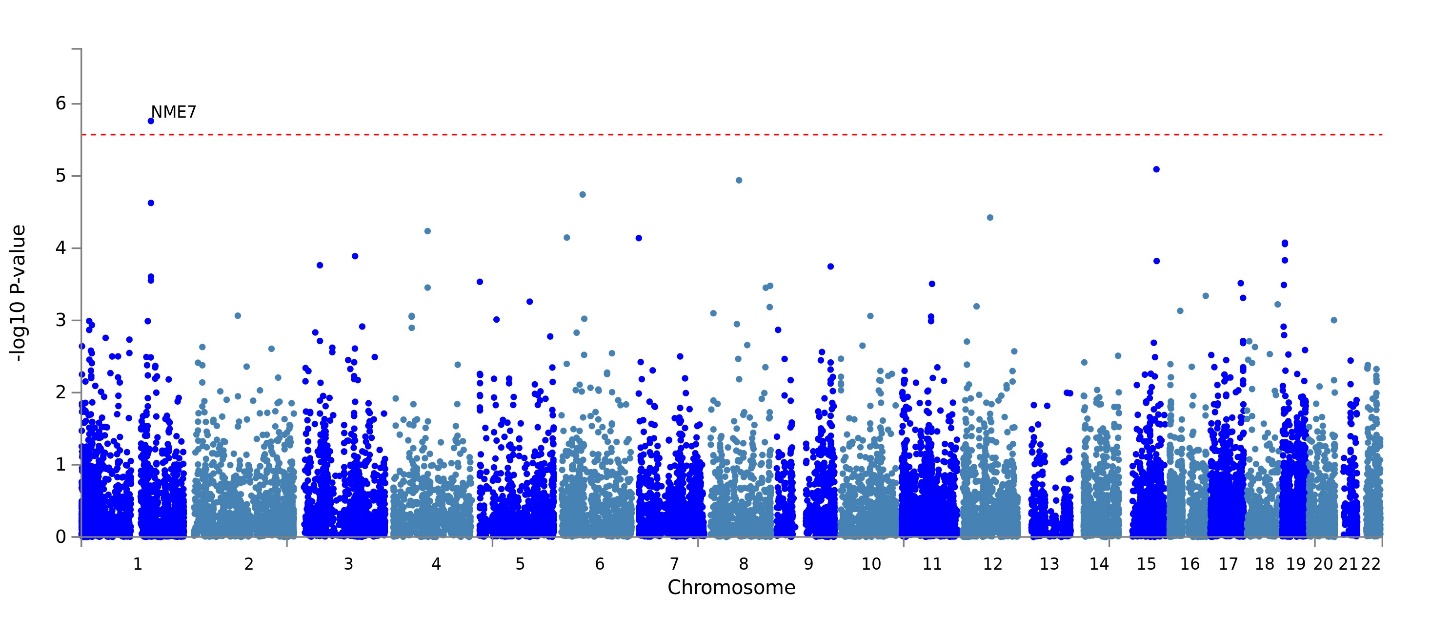


(b)


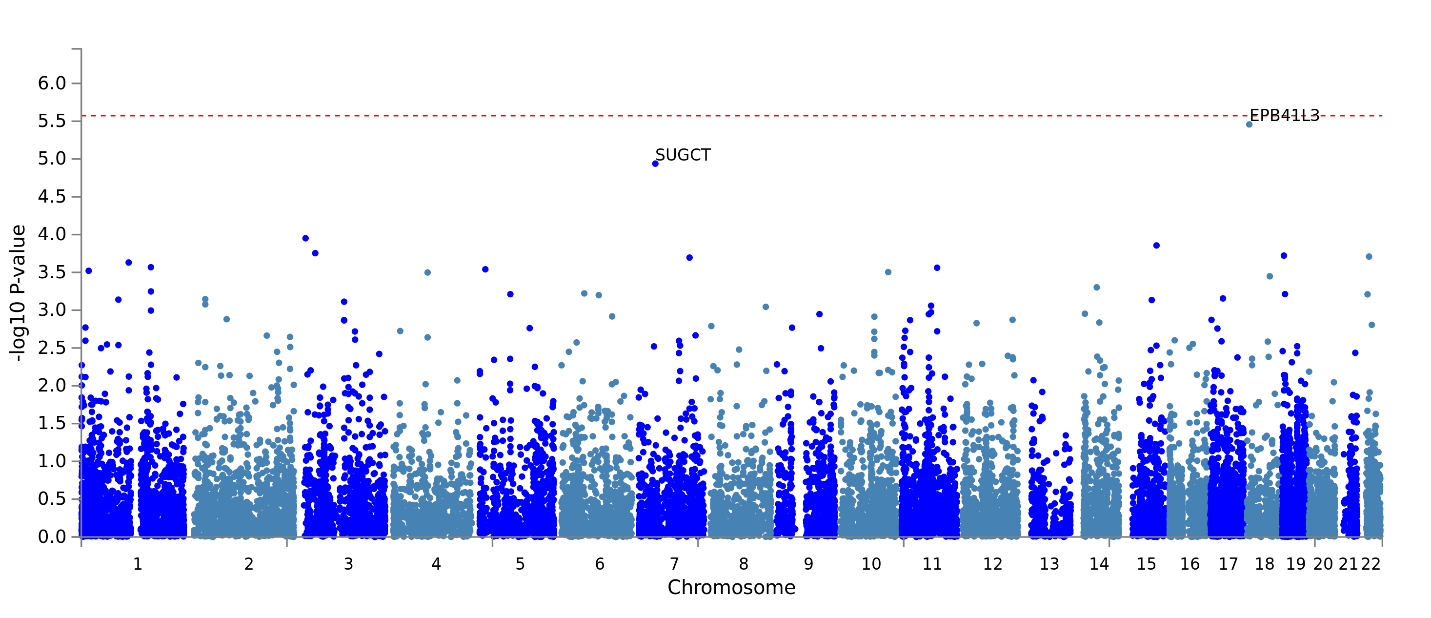


(c)


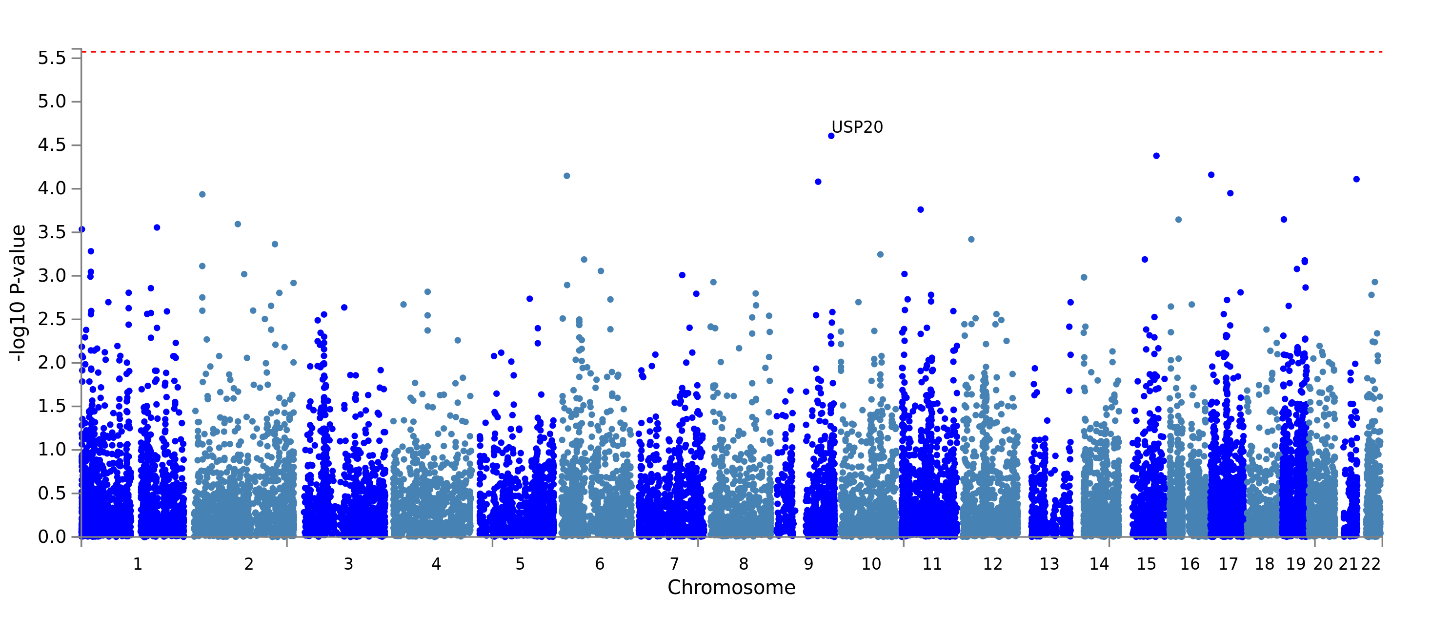


**Figure S5** Suggestive association of depression symptom PRS with esketamine treatment response outcome across different p-value thresholds (P_T_) in base GWAS (A) Responder status; (B) Percentage Change of MADRS at endpoint compared to baseline


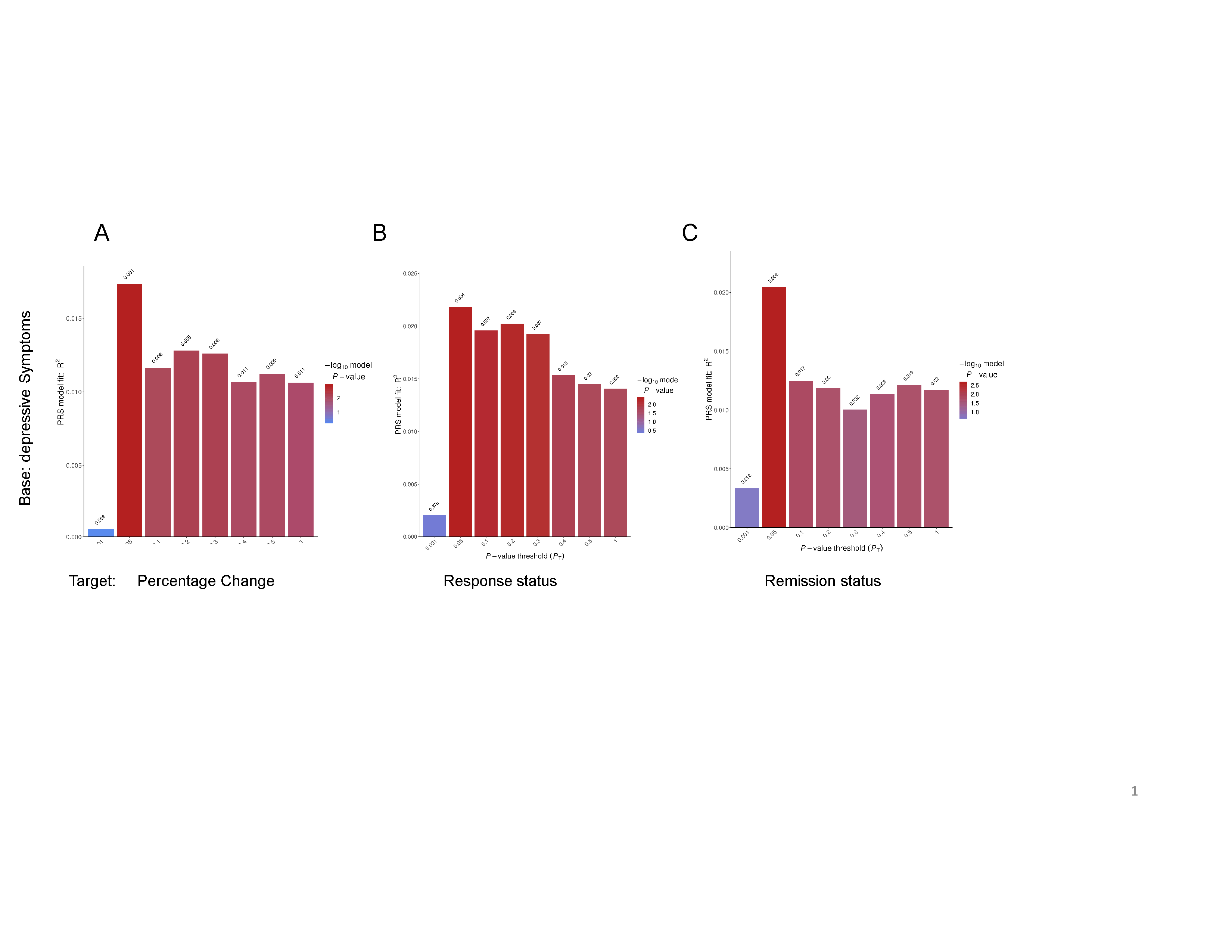


**Figure S6** Population substructure for the subjects retained in the GWAS analysis. (a) Principal components 1 vs 2; (b) Principal components 2 vs 3; (c) Principal components 3 vs 4

(a)


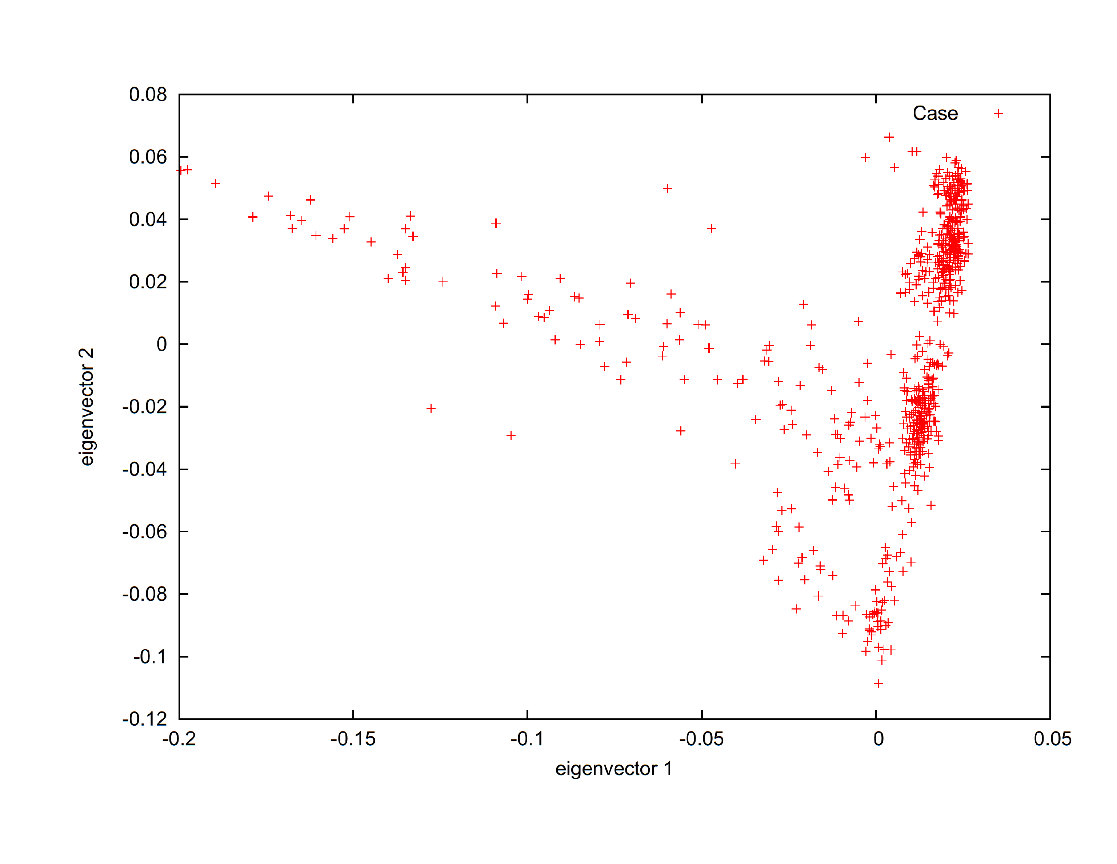


(b)

**
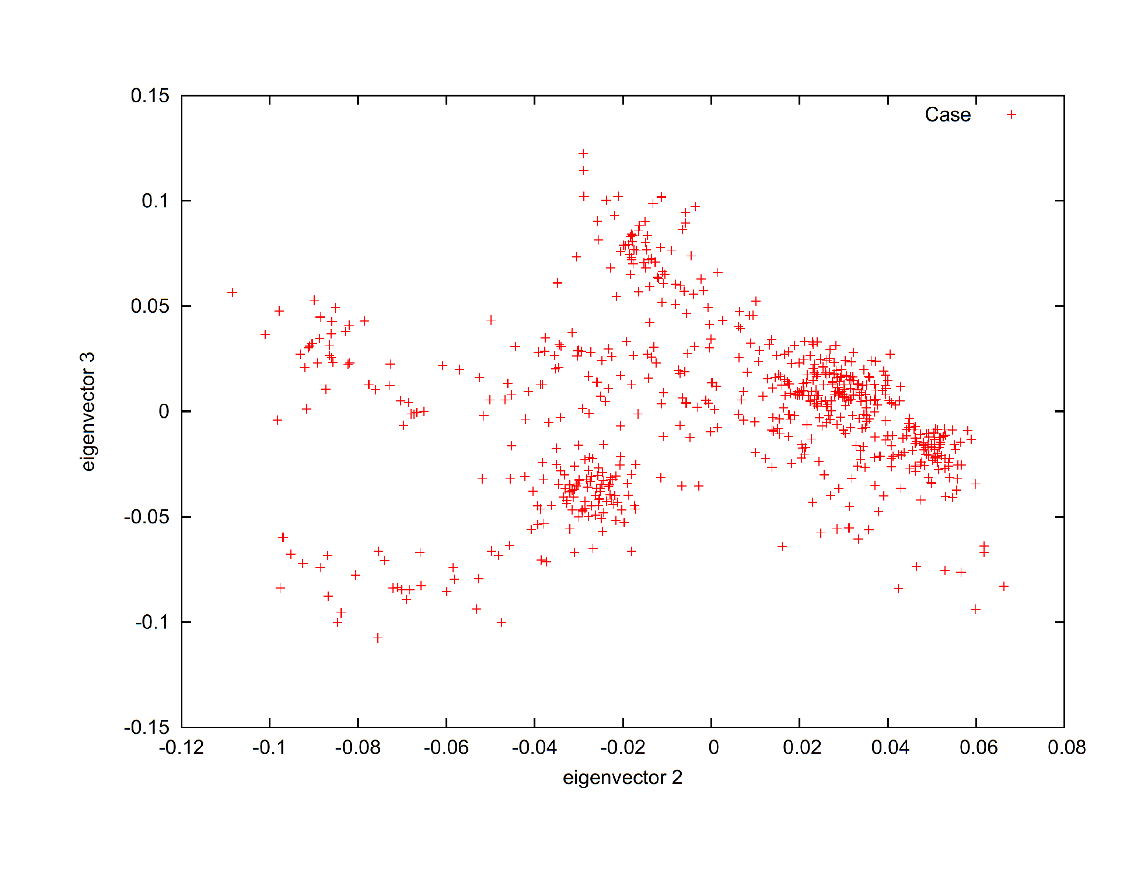
**

(c)

**
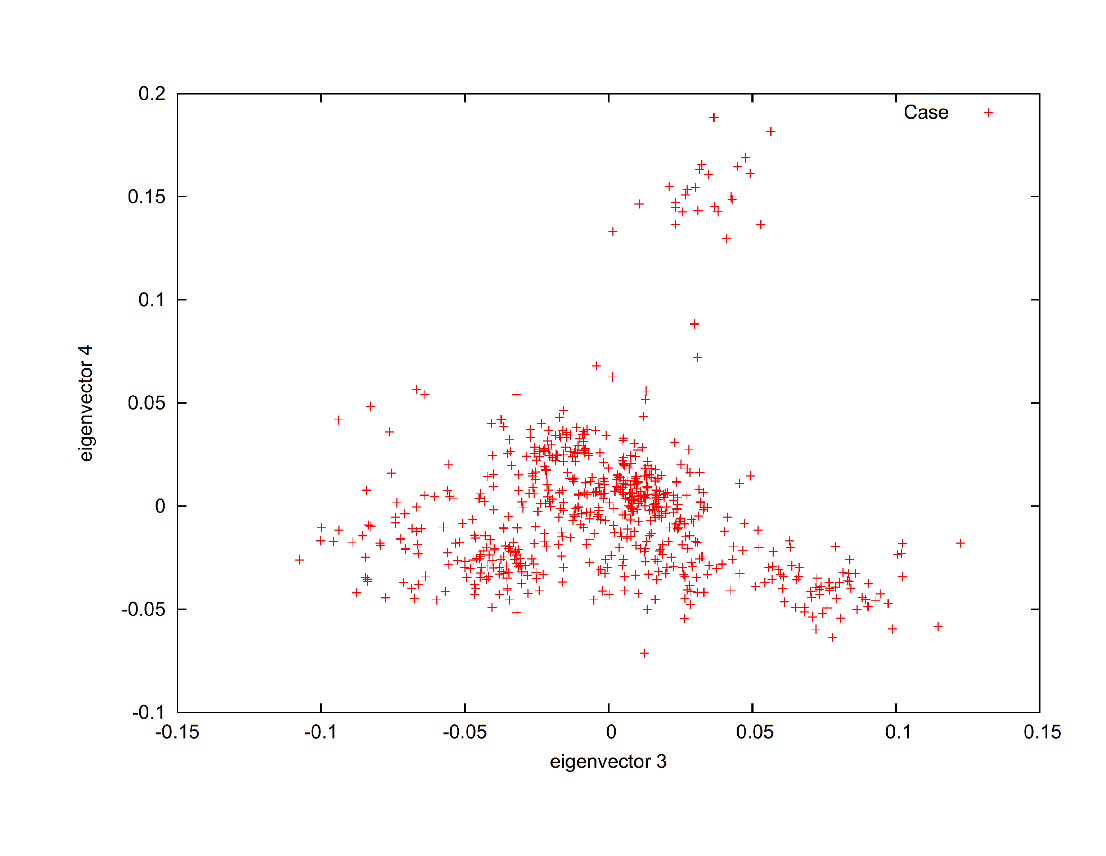
**

References

1. Wajs, E. *et al.* Esketamine Nasal Spray Plus Oral Antidepressant in Patients With Treatment-Resistant Depression: Assessment of Long-Term Safety in a Phase 3, Open-Label Study (SUSTAIN-2). *J Clin Psychiatry* **81**(2020).

2. Ochs-Ross, R. *et al.* Efficacy and Safety of Esketamine Nasal Spray Plus an Oral Antidepressant in Elderly Patients With Treatment-Resistant Depression-TRANSFORM-3. *Am J Geriatr Psychiatry* **28**, 121-141 (2020).

3. Purcell, S. *et al.* PLINK: a tool set for whole-genome association and population-based linkage analyses. *Am J Hum Genet* **81**, 559-75 (2007).

4. Patterson, N., Price, A.L. & Reich, D. Population structure and eigenanalysis. *PLoS Genet* **2**, e190 (2006).

5. Price, A.L. *et al.* Principal components analysis corrects for stratification in genome-wide association studies. *Nat Genet* **38**, 904-9 (2006).

6. Marchini, J., Howie, B., Myers, S., McVean, G. & Donnelly, P. A new multipoint method for genome-wide association studies by imputation of genotypes. *Nat Genet* **39**, 906-13 (2007).

7. Howie, B.N., Donnelly, P. & Marchini, J. A flexible and accurate genotype imputation method for the next generation of genome-wide association studies. *PLoS Genet* **5**, e1000529 (2009).

8. Marchini, J. & Howie, B. Genotype imputation for genome-wide association studies. *Nat Rev Genet* **11**, 499-511 (2010).

9. Howie, B., Marchini, J. & Stephens, M. Genotype imputation with thousands of genomes. *G3 (Bethesda)* **1**, 457-70 (2011).

10. Howie, B., Fuchsberger, C., Stephens, M., Marchini, J. & Abecasis, G.R. Fast and accurate genotype imputation in genome-wide association studies through pre-phasing. *Nat Genet* **44**, 955-9 (2012).

11. de Leeuw, C.A., Mooij, J.M., Heskes, T. & Posthuma, D. MAGMA: generalized gene-set analysis of GWAS data. *PLoS Comput Biol* **11**, e1004219 (2015).

12. Pruim, R.J. *et al.* LocusZoom: regional visualization of genome-wide association scan results. *Bioinformatics* **26**, 2336-7 (2010).

13. Watanabe, K., Taskesen, E., van Bochoven, A. & Posthuma, D. Functional mapping and annotation of genetic associations with FUMA. *Nat Commun* **8**, 1826 (2017).
